# Nationwide study on SARS-CoV-2 transmission within households from lockdown to reopening, Denmark, 27 February 2020 to 1 August 2020

**DOI:** 10.2807/1560-7917.ES.2022.27.6.2001800

**Published:** 2022-02-10

**Authors:** Frederik Plesner Lyngse, Carsten Kirkeby, Tariq Halasa, Viggo Andreasen, Robert Leo Skov, Frederik Trier Møller, Tyra Grove Krause, Kåre Mølbak

**Affiliations:** 1 Department of Economics & Center for Economic Behaviour and Inequality, University of Copenhagen, Copenhagen, Denmark; 2 Danish Ministry of Health, Copenhagen, Denmark; 3 Statens Serum Institut, Copenhagen, Denmark; 4 Department of Veterinary and Animal Sciences, Faculty of Health and Medical Sciences, University of Copenhagen, Copenhagen, Denmark; 5 Department of Science, Roskilde University, Roskilde, Denmark

**Keywords:** COVID-19, SARS-CoV-2, transmission, household, Attack rate, AGE

## Abstract

**Background:**

The COVID-19 pandemic is one of the most serious global public health threats of recent times. Understanding SARS-CoV-2 transmission is key for outbreak response and to take action against the spread of disease. Transmission within the household is a concern, especially because infection control is difficult to apply within this setting.

**Aim:**

The objective of this observational study was to investigate SARS-CoV-2 transmission in Danish households during the early stages of the COVID-19 pandemic.

**Methods:**

We used comprehensive administrative register data from Denmark, comprising the full population and all COVID-19 tests from 27 February 2020 to 1 August 2020, to estimate household transmission risk and attack rate.

**Results:**

We found that the day after receiving a positive test result within the household, 35% (788/2,226) of potential secondary cases were tested and 13% (98/779) of these were positive. In 6,782 households, we found that 82% (1,827/2,226) of potential secondary cases were tested within 14 days and 17% (371/2,226) tested positive as secondary cases, implying an attack rate of 17%. We found an approximate linear increasing relationship between age and attack rate. We investigated the transmission risk from primary cases by age, and found an increasing risk with age of primary cases for adults (aged ≥ 15 years), while the risk seems to decrease with age for children (aged < 15 years).

**Conclusions:**

Although there is an increasing attack rate and transmission risk of SARS-CoV-2 with age, children are also able to transmit SARS-CoV-2 within the household.

## Introduction

In late 2019, increased numbers of severe respiratory infections caused by the severe acute respiratory syndrome coronavirus 2 (SARS-CoV-2) were reported in the Wuhan province, China [1]. Since its emergence, the virus has spread rapidly throughout all five continents affecting millions of people [2] and causing economic losses [3]. During the early pandemic, the basic reproduction number was estimated to range from 2.1 to 4.7 in various studies that used different methods [4], revealing a high transmission potential. Person-to-person transmission is a major mode of transmission of SARS-CoV-2, including transmission through aerosols or airborne droplets [5,6]. Quantifying the transmission risk in different settings is essential for improving our understanding of viral transmission dynamics, to implement effective preventive measures, to minimise economic damage and to avoid overloading the healthcare system. Close person-to-person contact is a main risk factor for transmission, therefore the household is a major setting for virus transmission [6-9]. Furthermore, infection control and isolation are challenging in the potentially crowded household domain. Quantifying the extent of transmission within the household can help improve our understanding of the effects of implementing quarantine for household members, physical distancing and improved hygiene. These estimates are also useful in constructing reliable prediction models for the spread of SARS-CoV-2 from households to the community.

Data from contact tracing and monitoring of individuals have been used to investigate household transmission of SARS-CoV-2 [10-14]. Contact tracing is laborious and requires a large amount of resources when there are many new cases, as in the case of the coronavirus disease (COVID-19) pandemic. Thus, studies have been limited to include a maximum of a few hundred primary cases, selected mainly because of a history of hospitalisation or clinical disease [14]. The relatively small sample sizes as well as the selection and recall bias caused by contact tracing and different definitions of close contacts may limit the generalisability of these studies. In Denmark, residents have access to tax-based universal health insurance and SARS-CoV-2 testing is free of charge. Denmark ramped up testing capacity quickly during the first wave of the COVID-19 pandemic, resulting in widespread testing. In addition, Denmark has comprehensive social insurance and sick leave linked to SARS-CoV-2 infection is fully reimbursed by the state. Thus, neither financial reasons nor accessibility were major obstacles to obtaining a test, except during the initial phase of the epidemic.

**Figure 1:**
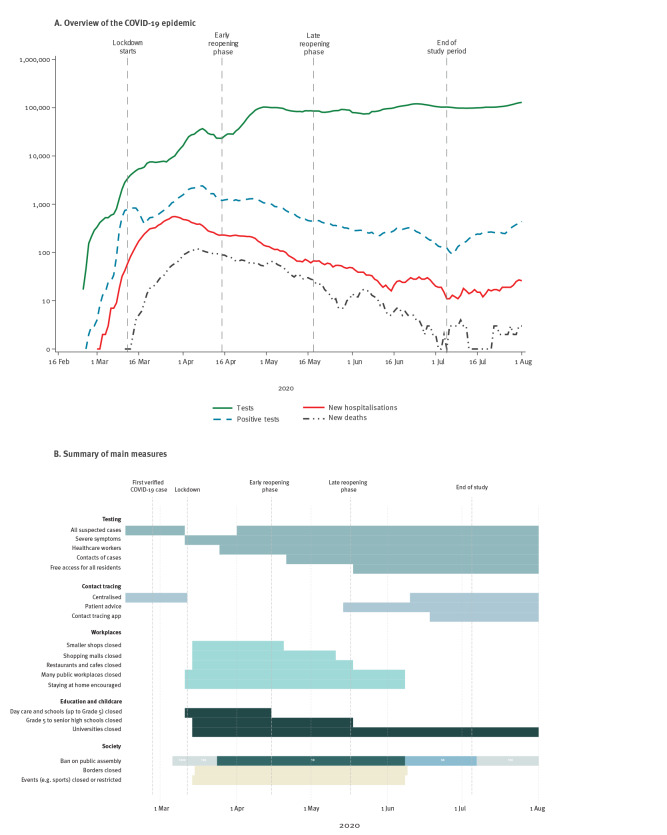
Overview of the COVID-19 epidemic in Denmark, 16 February 2020–1 August 2020

On 27 February 2020, the first case of SARS-CoV-2 was diagnosed in Denmark [15]. Shortly thereafter, the number of cases began to rise with an estimated effective reproduction rate of around 2.5 [16]. On 11 March 2020, a comprehensive lockdown of the public sector was implemented by the government. During lockdown, educational institutions were kept closed and public sector workers with non-essential functions stayed at home (Figure 1B). The availability of children’s day care (for children aged 1–5 years) was limited to children of employees in essential functions, such as doctors, nurses and police. Employees in the private sector were encouraged to stay at home if possible and international travel was minimised by closing the borders, except for essential activities. Following these measures, a reduction in the numbers of newly confirmed COVID-19 cases, COVID-19-related hospitalisations and deaths was observed in the second week of April 2020. In light of this, a partial easing of lockdown measures began on 15 April 2020, hereafter referred to as the early reopening phase. In the early reopening phase, children’s day care and school classes up to Grade 5 (students aged 6–10 years) reopened. As the situation continued to improve, further easing of the lockdown took place on 18 May 2020, hereafter referred to as the late reopening phase. In the late reopening phase, school classes for Grades 5–10 (students aged 11–16 years) and higher educational institutions reopened, along with restaurants and smaller bars with physical distancing measures in place.

COVID-19: coronavirus disease.

A. Key indicators of the COVID-19 epidemic presented: number of tests, positive test results, hospitalised cases, deaths during the early stage of the epidemic, the lockdown period and the early and late reopening phases.

B. Main measures for controlling the COVID-19 epidemic over time presented: test and contact tracing strategy, restrictions on public and private sector workplaces, education and childcare institutions as well as in the society in general.

Testing capacity increased throughout the COVID-19 epidemic and the number of tests remained stable from late April 2020 until the beginning of July 2020 (Figure 1A). At the beginning of the epidemic, all suspected cases of COVID-19 were tested and their contacts traced. However, because of the increasing number of cases and test capacity constraints, on 11 March 2020 the test strategy changed so that only cases with severe symptoms (i.e. patients admitted to hospital) were tested and contact tracing, which was overwhelmed, was halted.

From May 2020, testing became generally accessible, so that all residents could obtain a test without a referral (Figure 1B). In the late reopening phase, systematic contact tracing was also resumed.

The aim of this study was to investigate SARS-CoV-2 transmission in Danish households during the early stages of the COVID-19 pandemic.

## Methods

### Register data

In the current study, we used Danish administrative register data. All residents in Denmark have a unique personal identification number that allows an accurate linkage of information across different registers at the individual level. All microbiological data in Denmark is registered in the Danish Microbiology Database, from where we obtained individual level data on all national tests for SARS-CoV-2 from 27 February 2020 (the day of the first positive test in Denmark) to 1 August 2020.

Information on the reason for being tested, such as symptoms and potential contact with infected persons, was not available. We obtained information on sex, age and home address for all individuals living in Denmark from the Danish Civil Registration System [17].

### Data linkage and case definition

We constructed households by linking all individuals living at the same address and only considered households with six or fewer members in order to exclude institutions.

Thus, six single apartments in an apartment block were counted as six independent households. The dataset captured 98.3% per cent of the Danish population, residing in 2,730,296 households of which 1,066,524 included one person only. These single-person households were not included in the present study. Person-level data, which included information on the test result and date and time of sampling as well as time of the result, were linked to individuals within households. For each household, we identified the first individual with a positive RT-PCR test for SARS-CoV-2, who was defined as the primary case and referred to as such throughout this paper. We considered all subsequent SARS-CoV-2 tests from other members in the same household as tests taken in response to the primary case. We defined secondary cases as those who had a positive test within 14 days of the primary case testing positive for SARS-CoV-2. Primary examination of the data revealed that this cut-off provided a stable proportion of potential secondary cases. In addition, we assumed that the secondary household members were infected by the household primary case, although some of these secondary cases could represent co-primary cases. A longer cut-off time period could result in misclassification of cases among household members with the source of secondary infections lying elsewhere.

### Robustness of estimates over time

The test strategy and capacity changed over the study period (Figure 1). Since this could potentially bias our results in relation to time, we separated the data into three datasets representing three time periods based on the test date of the primary case. We performed analyses separately on these datasets. The defined periods were: (i) lockdown, 12 March to 14 April 2020; (ii) early reopening phase, 15 April to 17 May 2020; and (iii) late reopening phase, 18 May to 5 July 2020.

### Statistical analyses

To determine the probability that an additional household member would test SARS-CoV-2-positive by PCR after the primary case in the household tested positive, we used all records for potential secondary cases within the household. We defined potential secondary cases as all individuals living in the same household, excluding the primary case.

We defined the attack rate as the proportion of additional household members that tested positive, whereas the transmission risk was the proportion of secondary cases for each primary case. We did not exclude any potential co-primary cases in the main analysis. We further conducted a sensitivity analysis for the robustness of the cut-off period of transmission from the primary case to potential secondary cases.

We used SAS version 9.4 (SAS Institute, Cary, United States) to manage and analyse the data. In order to reach sufficient sample sizes, we separated all records into age groups of 5-year intervals.

#### Testing dynamics

To investigate the testing dynamics, we took an event study approach [18]. Following this method, we used the date of diagnosis of the primary case in each household as an event and observed all other household members from 5 days before until 14 days after the event. In the case of two or more primary cases detected on the same date, we randomly assigned one of them as the primary case. We estimated the probability of being tested (*β_τ_*) for each day relative to the first positive test result within the household, using the following equation:


$$y_{i,t}=\sum_{\tau =-5}^{14}\left(I_{\tau \geq t}\times \beta_{\tau }+\varepsilon_{i,t}\right)\;\;\;\;\;\;\;\left(Equation\;1\right),$$

where *y_i,t_* is a binary variable for individual *i* being tested at time *t* and *τ* is days relative to the date of the primary case’s positive test result. Indicators for time since the primary case’s positive test result are denoted by *I_τ_*_=_ *_t_* while *β_τ_* represents parameters estimating the probability of being tested on day *τ* relative to receiving the primary case’s test result in the household. The error term, clustered on the household (event) level, is denoted by *ε_i,t_*. We used the same equation to estimate the probability of *y* testing positive conditional on being tested.

Furthermore, to estimate the proportion of potential secondary cases that had been tested on day *τ* or previously, we estimated the absorbing probability using the following equation:


$$y_{i,t}=\sum_{\tau =0}^{14}\left(I_{\tau \geq t}\times \beta_{\tau }+\varepsilon_{i,t}\right)\;\;\;\;\;\left(Equation\;2\right),\;\;$$

where *I_τ_*_≥_ *_t_* = 1 if individual *i* was tested on day *τ* or previously and zero otherwise. We also used the same equation to estimate the probability of *y* ever being tested positive.

In the results section, we focused only on the testing dynamics during the late reopening phase, when test capacity was stable. We also performed the analysis on the two other periods, lockdown and early reopening phase (data not shown).

#### Proportion of total positive cases originating from households

To investigate the proportion of positive tests originating from households, we defined new cases that lived in a household with another case who tested positive within the preceding 14 days as a case originating from the household domain. We used a 7-day rolling average in order to take account of variation in testing rates across the weekdays.

#### Attack rate

To estimate the attack rate, we calculated the proportion of potential secondary household members who received a positive test within 14 days after the test date of the primary case. We estimated attack rates using the following equation:


*y_i,t_* = *β*_0_ + *γPeriod* + *φFemale* + *δFemale* × *Period* + *ε_i,t_,* (Equation 3),

where *y_i,t_* = 1 if the individual had a positive test within the 14 days after the primary case, and zero otherwise. A vector of fixed effects for the three periods is denoted by *γ*. Female is a binary variable for sex. The 14-day attack rate is measured by *β_0_* while *ε_i,t_* denotes the error term, clustered on the household (event) level.

#### Age-structured attack rate and transmission risk

To investigate the age structure of the attack rate for potential secondary cases and transmission risk from primary cases, we used a non-parametric approach. We separated the data into 5-year age groups. We estimated the attack rate using the following equation:


*y_i,t_* = *α* × *AgeGroup_s_* + *ε_i,t_,* (Equation 4),

where *y_i,t_* = 1 if the potential secondary case *i* had a positive test within the 14 days after the test date of the primary case and zero otherwise. The *AgeGroup_s_* represents 5-year age groups of the potential secondary cases, *α* is a vector that measures the age-structured attack rate and *ε_i,t_* denotes the error term, clustered on the household (event) level.

We estimated the transmission risk using the following equation:


*y_i,t_* = *β* × *AgeGroup_p_* + *ε_i,t_,* (Equation 5),

where *AgeGroup_p_* represents 5-year age groups of the primary cases and *β* is a vector that measures the age-structured transmission risk.

We estimated the age-structured interaction between the attack rate and transmission risk using the following equation:


*y_i,t_* = *γ*×*AgeGroup_s_* × *AgeGroup_p_* + *ε_i,t_,* (Equation 6),

where *γ* is a vector that measures the age-structured interaction between attack rate and transmission risk.

To quantify the effect of age on attack rate, we estimated the approximate linear relationships between attack rate and age using the following equation:


*y_i,a_* = *α*_0_ + *βAge* + *φPeriod* + *δAge* × *Period* + *πFemale* × *Period* + *ε_i,a_,* (Equation 7),

where *β* measures the probability of being infected as a linear function of age, *φ* is a vector of fixed effects for each time period (see ‘Robustness of estimates over time’ section), *δ* measures the differential age gradient for each period and *π* measures the effect of sex for each period.

In order to investigate the potential difference between male and female cases, we explored the age-dependent attack rate separately for each sex. We also separated the data into households where the primary cases were children (under 15 years of age) and adults (aged 25 years or older), while individuals aged 15–24 were excluded. These thresholds were chosen to ensure that the primary cases were either children or adults.

#### Household size structured attack rate

We estimated the attack rate stratified by the number of household members for households with two to six members. Furthermore, we estimated the proportion of households with N number of positive cases, conditional on the size of the household being greater than or equal to N with a maximum household size of six.

### Ethical statement

This study was conducted on administrative register data. According to Danish law ethical approval is not needed for such research. All data management and analyses were carried out on the Danish Health Data Authority’s restricted research servers with project number FSEID-00004942.

## Results

### Descriptive statistics

In total, we obtained positive test results from 6,782 household primary cases and 1,904 positive secondary cases (Table 1).

**Table 1:** Summary statistics of the study population by case status and phase of control measures taken, Denmark, 12 March–5 July 2020 (n = 21,015 individuals; 6,782 households)^a^

Case status	Lockdown 12 Mar–14 Apr 2020	Early reopening phase 15 Apr–17 May 2020	Late reopening phase 18 May–5 Jul 2020	Total
Primary cases	3,612	2,180	990	6,782
Potential secondary cases	7,386	4,621	2,226	14,233
Tested secondary cases	1,836	2,952	1,821	6,609
Positive secondary cases	807	726	371	1,904
PCR-tests (%)				
Testing rate	0.25	0.63	0.82	0.46
Positivity rate	0.11	0.16	0.17	0.13

^a^ Only primary cases living with 1–5 other persons were included as primary cases in this table. Potential secondary cases are the total number of persons sharing a household with a primary case, excluding the primary case.

We looked at the probability of being tested during the study period in the 5-year age groups and found that all age groups were being tested, although generally with lower probability for children (Supplementary Figure S1). The increase in testing capacity over time was approximately equally distributed across ages. In late June 2020, we saw an increase in the probability of children being tested.

In addition, we looked at the age distribution of primary cases compared with the overall Danish population and found that there were proportionally fewer children (below the age of 15 years) who were primary cases than in the total population and that household sizes of the primary cases generally matched the household sizes of the population (data not shown).

### Testing dynamics

**Figure 2:**
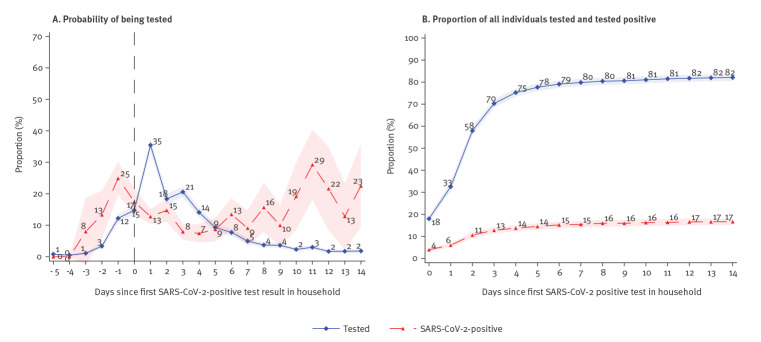
Dynamics of SARS-CoV-2 testing, Denmark, 18 May–5 July 2020 (n = 3,216 individuals; 990 households)

Figure 2A, shows that after receiving a positive test result in the household (*t* = 0), 35% (788/2,226) of potential secondary cases were tested the day after the positive test result (*t* = 1) of the primary case was available. Of the 788 performed tests, 779 had a conclusive (positive/negative) result and 13% (98/779) were positive. On the day preceding the test result (*t* =  − 1), 12% (271/2,226) were tested and 25% (65/264) of these tests were positive (and six were inconclusive).

SARS-CoV-2: severe acute respiratory syndrome coronavirus 2.

A. *t* = 0 denotes the time of receiving the first positive test result in a household. On the day preceding the result (*t* =  − 1) 12% were tested and 25% of these tested positive. The day after the households received their first positive test result (*t* = 1), 35% were tested and 13% of these tested positive.

B. *t* = 0 denotes the time the first positive test was taken (not when the test result was given). 18% of all potential secondary cases were tested on the same day as the primary case (*t* = 0) and 4% of all potential secondary cases tested positive. This implies a 22% probability of testing positive conditional on being tested on the same day as the primary case. Fourteen days after the day of the first positive test, 82% had been tested and 17% tested positive.

Data shown refers to the late reopening phase (18 May 2020 to 5 July 2020). Shaded areas show the 95% confidence bands clustered on household level.

Figure 2B shows the proportion of individuals that were tested and those that tested positive daily up to 14 days after the primary case was tested (*t* = 0). Here, 18% (400/2,226) of potential secondary cases were tested on the same day as the primary case and 4% (87/2,226) of potential secondary cases tested positive on that day. Within 14 days after the primary case was tested, 82% (1,827/2,226) of the potential secondary cases were tested and 17% (371/2,226) tested positive, implying an attack rate of 17%.

There were 4% who tested positive on the same day (*t* = 0) as the primary case. These may represent co-primary cases and therefore may not represent household cases. Under this assumption, in order to estimate the attack rate, one should exclude these cases by subtracting 4 percentage points (pp) from 17%, thereby resulting in an attack rate of 13%. Similarly, cases detected within 1 day of the primary case may represent co-primary cases (*t* ≤ 1). This leaves an attack rate of 11% (by subtracting 6 pp from 17%) (the Supplement provides a robustness analysis on the definition of co-primary cases).

### Proportion of total positive cases originating from households

**Figure 3:**
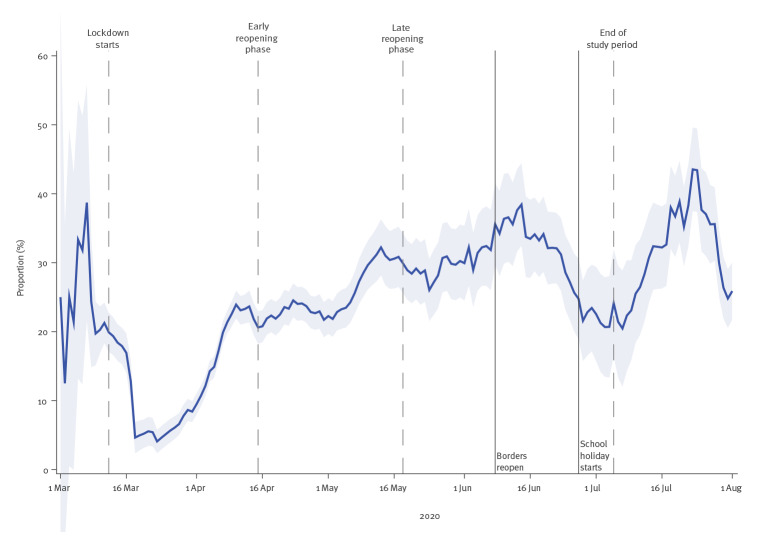
Proportion of total SARS-CoV-2-positive cases originating from households, Denmark, 1 March–1 August 2020 (n = 13,082)

Figure 3 shows the proportion of positive tests originating from households within the 14 days following the day when the primary case tested positive. After a rapid decrease immediately after lockdown, there was an increasing proportion of new cases originating from households during lockdown and a decreasing proportion after reopening of the borders.

SARS-CoV-2: severe acute respiratory syndrome coronavirus 2.

This figure shows the proportion of positive tests originating from households, as defined by new cases that live in a household with another case that tested positive within the preceding 14 days. The figure shows a 7-day moving average, while the shaded area shows the 95% confidence bands with standard errors clustered on the individual level.

### Age-structured attack rate

**Figure 4:**
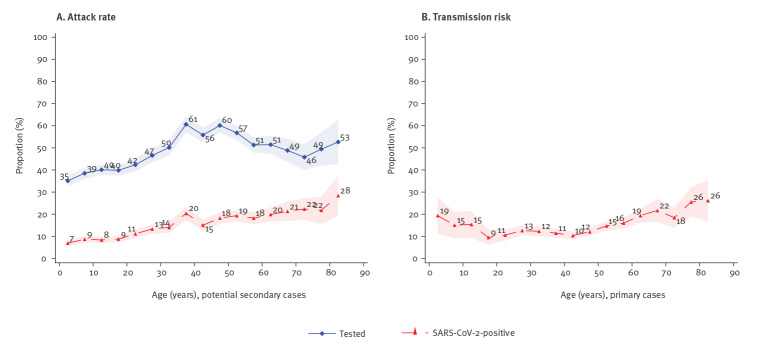
Age-structured attack rate (A) and transmission risk (B) of SARS-CoV-2 transmission in households, Denmark, 12 March–5 July 2020 (n = 21,015 individuals; 6,782 households)

We found an approximately linearly increasing relationship between age and attack rate. Figure 4A, shows the probability of having a test and the probability of having a positive test (unconditional on being tested) across 5-year age groups. The shaded areas show the 95% confidence bands. While Figure 4A shows an increasing attack rate with age, Figure 4B shows the transmission risk from primary cases by age group, i.e. the probability of infecting others. Figure 4B shows an increasing risk with age for adults, while the risk seems to decrease with age for children.

SARS-CoV-2: severe acute respiratory syndrome coronavirus 2.

A. This figure shows the probability of having a test (blue) and the probability of having a positive test (red) (unconditional on being tested) across 5-year age groups.

B. This figure shows the transmission risk from primary cases by age group, i.e. the probability of infecting others.

The shaded areas show the 95% confidence bands clustered on the household level.

As Figure 4A shows an approximate linear relationship between attack rate and age, we estimated the linear relationship using Equation 7 (Table 2 Model I). The results show that individuals had a baseline risk of 9.4% of testing SARS-CoV-2-positive. The risk increased by 0.24 percentage points for each year of age. Thus, a 10-year-old had a risk of 11.8%, a 30-year-old had a risk of 19.0% and a 60-year-old had a risk of 33.4%. The estimates were robust to different specifications, including period and sex covariates (Table 2 Model II and Model III).

**Table 2:** Regression estimates of SARS-CoV-2 attack rate by age, Denmark, 12 March–5 July 2020 (n = 21,015; 6,782 households)

Variables	Model I		Model II		Model III	
	Estimate	SE	Estimate	SE	Estimate	SE
Intercept	9.39	1.07^a^	10.12	1.62^a^	8.58	1.82^a^
Age	0.24	0.01^a^	0.21	0.04^a^	0.22	0.04^a^
Lockdown	6.00	1.07^a^	− 8.20	1.72^a^	− 7.71	1.93^a^
Early reopening	− 0.79	1.19	0.66	1.93^a^	0.32	2.13
Late reopening	Ref.		Ref.		Ref.	
Age × lockdown^b^	–^c^		0.07	0.05	0.07	0.05
Age × early reopening^b^	–^c^		− 0.05	0.05	− 0.05	0.05
Female	–^c^		–^c^		2.82	1.61
Female × lockdown^b^	–^c^		–^c^		− 0.69	1.75
Female × early reopening^b^	–^c^		–^c^		1.09	1.09
Number of households	6,782		6,782		6,782	
Number of observations	14,220		14,220		14,220	

SARS-CoV-2: severe acute respiratory syndrome coronavirus 2; SE: standard error.

^a^ p < 0.01.

^b^ The x denotes the interaction between the two variables.

^C^ Not included in the model.

Standard errors clustered on the household level.

We stratified attack rates conditional on the primary case being a child or an adult. When the primary case was a child (under 15 years of age), the attack rate peaked in adults in their early 30s, presumably the children's parents. This seemed uniform (with large standard errors) across the age of the other potential secondary cases (Supplementary Figure S2A). When the primary case was an adult (aged 25 years or older), the attack rate increased (linearly) with age (Supplementary Figure S2B).

We further investigated the interaction between the attack rate and transmission risk for each combination of age groups. In general, the probability for potential secondary cases of being tested was highest when the primary case was a child (under 15 years of age). The probability of being tested was also high when the age difference between the primary case and the potential secondary case was small. (Supplementary Figure S3A) The attack rate was highest when the primary and potential secondary cases were above 60 years of age. When children were the primary cases, an increased attack rate was observed for all age groups. (Supplementary Figure S3B)

We found no difference in the attack rates stratified by sex (Supplement SB1).

### Proportion of cases by household size

**Figure 5:**
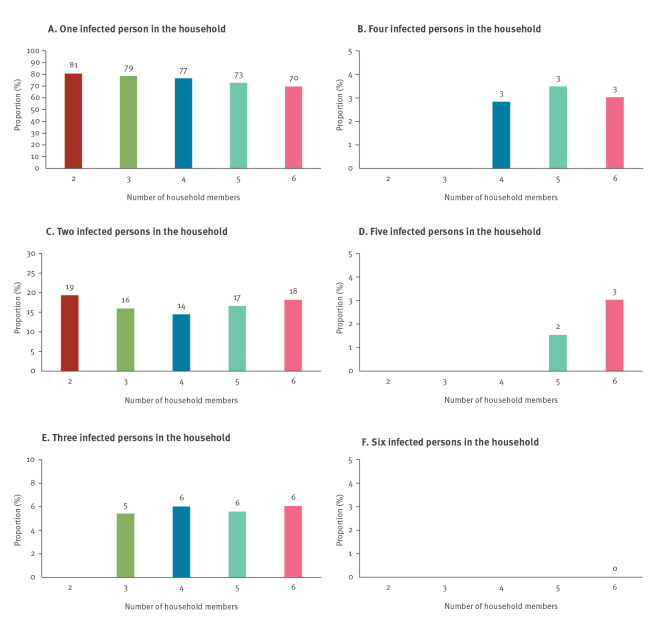
Proportion of SARS-CoV-2 cases by household size, Denmark, 12 March–5 July 2020 (n = 23,134 individuals; 6,782 households)

Figure 5 shows the proportion of cases by the number of household members for households with two to six members. For instance, in households with two members (red), 81% (2,367/2,935) of the households had one positive case, while 19% (568/2,935) had two positive cases. In households with three members (green) 79% (1,117/1,421) of the households had one case, 16% (227/1,421) had two cases, and 5% (77/1,421) had three cases (Supplementary Table S3). This implies that in a three-person household, 79% had an attack rate of 0% (0 secondary cases from two potential cases), 16% had an attack rate of 50% and 5% had an attack rate of 100%.

SARS-CoV-2: severe acute respiratory syndrome coronavirus 2.

The figure shows the proportion of cases by the number of household members for households with two to six members. For instance, in a household with two members (red), 81% of the households had only one positive case, while 19% had two positive cases. Similarly, in a household with three members (green) 79% of the households had one case, 16% had two cases, and 5% had three cases. Supplementary Table S3 presents the point estimates and standard errors.

Once a primary case was found within a household, the probability of at least one secondary case was 23% (1,556/6,782), regardless of household size, and consequently 77% (5,226/6,782) of the primary cases did not generate any additional cases (Supplementary Table S4). In particular, the primary cases had a 17% (1,136/6,782) probability of generating one additional case, 6% (221/3,847) of generating two additional cases, 3% (75/2,426) of generating three additional cases, and 2% (18/947) of generating four additional cases. This pattern was consistent regardless of the number of persons in the household, showing a transmission pattern that was exponentially decreasing with the number of members within the household. The pattern was consistent over the three phases of the epidemic examined here, indicating that it was not a result of the change in the testing strategy (data not shown).

### Robustness of estimates

The COVID-19 epidemic in Denmark changed over time, both because of changes in policy response (e.g. lockdown), and changes in test capacity and strategy (Figure 1). In this section, we investigated the robustness of the previously described results over the three defined periods of the epidemic. The analyses showed that despite the substantial changes in probability of being tested, the estimated attack rates were consistent throughout the epidemic (data not shown).

We investigated the sensitivity of the estimated attack rates to the definition of co-primary cases (Supplement SD). The analysis showed that the estimated age-structured attack rates did not change noticeably by excluding secondary cases found within the same day, 1 day or 2 days of the primary case.

## Discussion

We present results from a nationwide study that estimated household attack rates and transmission risks based on SARS-CoV-2 test data from an entire population during the early stages of the COVID-19 pandemic. We estimated an overall attack rate within households of 17%, ranging from 11% during lockdown to 16% during the early reopening phase and 17% during late reopening phase. This suggests that attack rates estimated early on in the Danish epidemic underestimated the true attack rate. This bias likely comes from the limited testing capacity at the beginning of the COVID-19 epidemic. These estimates are in line with the estimates from the literature; Madewell et al. [14] conducted a meta-analysis of household transmission of SARS-CoV-2 from 40 studies and found an overall attack rate of 18.8%, which is close to our estimate.

We studied the testing dynamics for COVID-19 and found that the probability of obtaining a test relative to a primary case positive test result within the household peaked on the day after the primary case received the result, when 35% of all potential secondary cases were tested and 13% of these were positive. Interestingly, 12% of the potential secondary household cases were tested 1 day preceding the test result of the primary case and 25% of these were positive. This could indicate that these individuals were tested for a reason other than the primary case result, e.g. for having symptoms themselves. The probability of being tested after a primary case in the same household increased from 18% on the same day as the primary case until it flattened out at around 80% on day 6. Of all secondary cases, 76% were found during the first 3 days after the primary case (the attack rate was 13% on day 3 and 17% on day 14). This highlights the importance of fast contact tracing, as most secondary cases were found in the first days after the primary case, which was also concluded by Moghadas et al. [19]. However, these cases could also represent co-primary cases.

The proportion of positive cases originating from households increased during the lockdown until the late reopening phase, when the borders reopened. Thereafter, it increased again after the school holidays started. In other words, the school holidays, which also included 3 weeks of annual leave for most parents with school-aged children, essentially functioned as another lockdown period because families tend to stay together during the holidays. We suggest that this may have contributed to a low incidence of community transmission over the summer of 2020. When the testing capacity was relatively stable (from late April 2020), the proportion of positive cases originating from households varied between 20% and 45% of total cases. This indicates that many cases originated within the household domain, and this should be taken into account when monitoring the epidemic as well as in national guidelines for COVID-19 prevention. Evidence from other countries also finds a substantial proportion of cases originating from households, such as in Israel where 67% of all SARS-CoV-2 infections were found to have originated at home [20]. Therefore, it is important to further investigate the dynamics of SARS-CoV-2 in homes, schools, workplaces and other places in the community in order to quantify their impacts on virus transmission and develop effective control measures.

We found a linear relationship between age and both the attack rate and transmission risk from primary cases. This suggests that susceptibility to infection increases with the age of the susceptible person. However, children (under 15 years of age) also had an elevated transmission risk, likely because of closer contact with parents, indicating that children may represent an overlooked risk. Our analysis did not include for example household density, i.e. that it is easier to keep a distance to an infected household member in a large house compared with a small apartment. Our findings correspond with existing literature, such as the study by Madewell et al. [14] which found that susceptibility to infection increased with age and a large seroprevalence study in Spain which also found an increasing linear relationship with age [21]. Similar findings were reported by Li et al. [13] and Bi et al. [22].

We further estimated the attack rates conditional on the primary case being a child or an adult. When the primary case was a child (under 15 years of age), the attack rate peaked in adults in their early 30s, presumably the children's parents, and seemed uniform across the age of the other potential secondary cases. When the primary case was an adult (aged 25 years and older), the attack rate increased linearly with the age of the potential secondary case. This suggests that transmission from children is constant, and depends on close contact with susceptible cases, whereas transmission from adults is more effective the older the potential secondary case is. One could think that if a child is sick, caregivers are likely to have even more close contact with the child and more so the younger the child is. The opposite may be true for adult cases, indicating that the susceptibility to COVID-19 increases with the age of a person, reflecting immunological properties. Although there is general agreement that transmission from and between children is not the main driver in this epidemic [23], transmission from sick children to parents in the household domain may represent a hitherto overlooked risk factor.

Since the testing capacity and strategy (and hence access to obtaining a test) has changed substantially over the course of the epidemic, the robustness of results is a primary concern when comparing positive test cases over the COVID-19 pandemic. We addressed this by dividing our sample into three periods with different testing capacities and found that the probability of obtaining a test did increase substantially across the periods. Our results were, however, relatively consistent over time, suggesting that the findings were not because of changing testing strategies. A potential bias is that the reopening increases activity in society and thereby increases the probability of secondary cases being infected in the community. However, the reopening is a direct consequence of a lower number of cases, hospitalisations, and deaths, which reduces overall community transmission risk. We believe that any community transmission will more likely result in co-primary cases. This also because contacts within the same household should isolate themselves from society after a confirmed case. Hence, the sensitivity analyses will take this misclassification into account.

There are no formal guidelines for defining co-primary cases in this type of study. Madewell et al. [14] provided a review on household transmission of SARS-CoV-2, however, most of the studies included did not describe how co-primary cases were handled. Several studies stated that they assumed all secondary cases were infected by the primary case, one study excluded secondary cases if they developed symptoms before exposure to the primary case and another study randomly selected one primary case as the source of infection. We addressed this important question by showing the sensitivity for different specifications. We found that the attack rate was strongly dependent on the definition. For instance, we showed that if one defines co-primary cases as individuals tested on the same day as the primary case, the secondary attack rate is reduced by 24%. Increasing the period for co-primary cases further caused the estimated attack rate to decrease even more. However, the definition did not change our overall results on the age-structured attack rates.

Mathematical modelling is a widely used tool for researchers to understand and predict the spread of disease and policy relies on obtaining proper results from these models when taking decisions such as choosing between keeping some parts of society open while closing others. The closure of childcare and schools has been a widespread measure adopted in most countries. The results from this study can be used as direct input in parameterising such mathematical models in terms of virus transmission at home. Furthermore, our estimates of the age-structured attack rate and transmission risk, as well as the interaction of these, are important inputs in mathematical models, for instance, for contact matrices between age groups [8,24].

When modelling the spread of COVID-19, many researchers assume that each contact has the same probability of transmission (conditional on time and distance of contact), i.e. a binomial process [11,25]. Our results, however, suggest that this should be modelled as a two-step procedure when simulating contacts between individuals. Firstly, it should be determined whether a case is infectious or not (i.e. a Bernoulli process), and secondly, conditional on being an infectious case, a binomial process should be used to represent actual transmission. This allows for more realistic replication of the transmission dynamics of the SARS-CoV-2 virus.

## Conclusions

Although there is an increasing attack rate and transmission risk of SARS-CoV-2 with age, children are also able to transmit SARS-CoV-2 within the household. Our results show important differences in transmission pattern with the age of both the primary case and the potential secondary cases within households. A large proportion of transmission was found to occur within households, highlighting the effectiveness of SARS-CoV-2 transmission and that preventative measures within households are needed in order to prevent transmission (these could include for example an increased focus on hygiene and distancing within the household and quick isolation of confirmed cases e.g. in designated facilities, i.e. ‘isolation hotels’). Moreover, monitoring the proportion of SARS-CoV-2-positive cases originating from households may be an important tool for public health authorities to measure community transmission.

Lastly, the method used in this study allows us to estimate the individual attack rates and transmission risks across different mutations, which is a primary concern for public health authorities.

## Supplementary Data

1110,000 Supplement
