# Supplementary material for: Nationwide study on SARS-CoV-2 transmission within households from lockdown to reopening, Denmark, 27 February 2020 to 1 August 2020
Source: Euro Surveill. 2022 Feb 10;27(6):2001800. doi: 10.2807/1560-7917.ES.2022.27.6.2001800 (PMC8832519; doi:10.2807/1560-7917.ES.2022.27.6.2001800)
Supplement: Supplement [file 20-01800_LYNGSE_Supplement.pdf]

This supplementary material is hosted by Eurosurveillance as supporting information alongside the article *"Nationwide study on SARS-CoV-2 transmission within households from lockdown to reopening, Denmark, 27 February 2020 to 1 August 2020"*, on behalf of the authors, who remain responsible for the accuracy and appropriateness of the content. The same standards for ethics, copyright, attributions and permissions as for the article apply. Supplements are not edited by Eurosurveillance and the journal is not responsible for the maintenance of any links or email addresses provided therein.

Supplementary Appendix A

Testing over the First Wave of the COVID-19 Epidemic in Denmark

Figure S1: Age Specific Probability of Obtaining a Test and a Positive Test

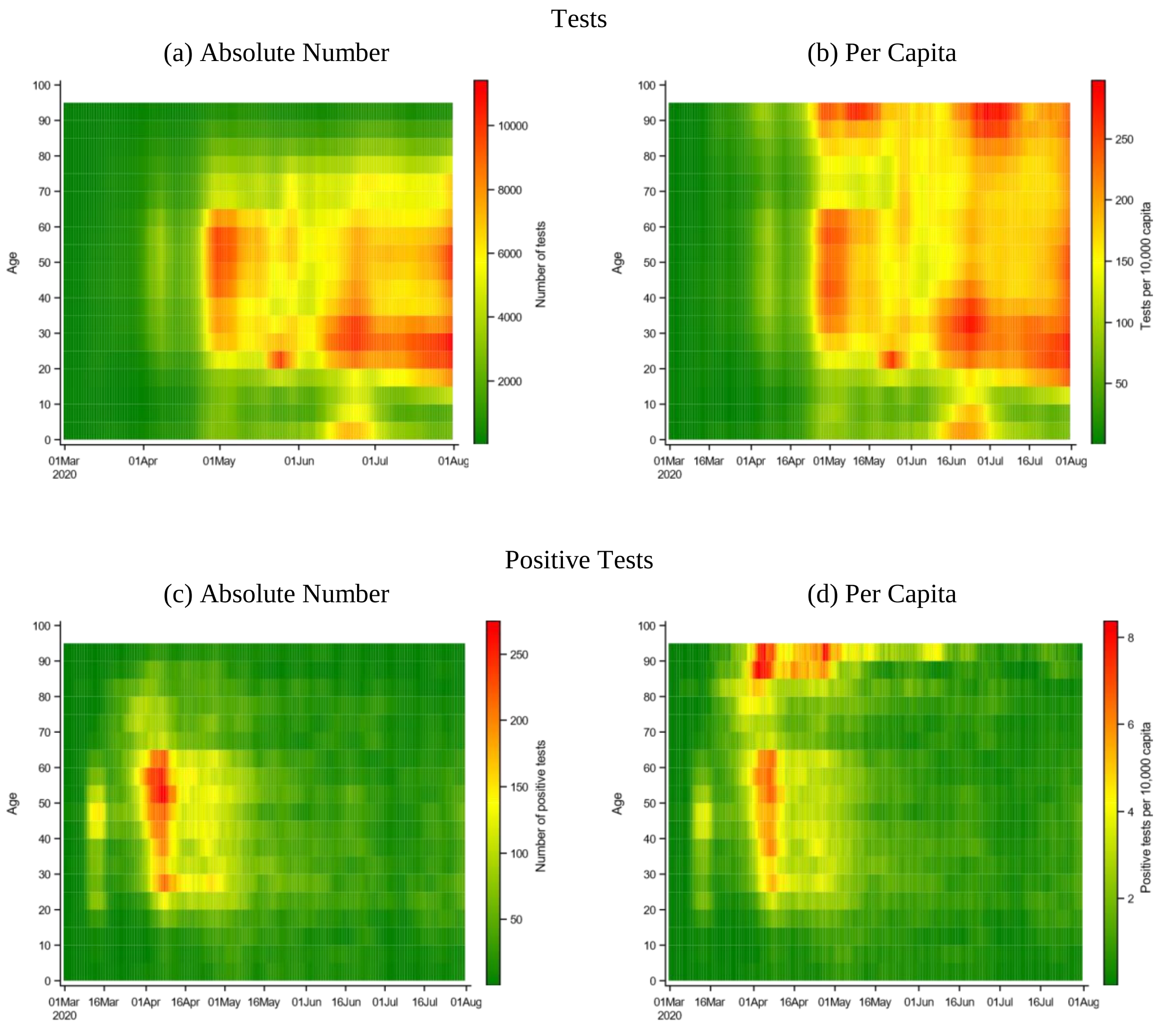

Notes: Age is 5-year-age groups. Numbers represent a 7-day-rolling sum. The figure is inspired by Marc Bevand, https://github.com/mbevand/florida-covid19-line-list-data

Table S1: Days From Test to Test Result

	Lockdown	Early Reopening	Late Reopening	Total
25 Percentile	0	0	1	0
50 Percentile	1	1	1	1
75 Percentile	1	2	1	1
95 Percentile	2	4	2	3
Mean	0.9	1.3	1.0	1.0
Number of tests	87,034	375,444	679,847	1,142,325

Notes: This table provides summary statistics on the number of days between the day of the test and the day of receiving the result (for all SARS-CoV-2 tests obtained in the study period).

Supplementary Appendix B

Age Structured Attack Rate

Figure S2: Age Structured Attack Rate by Age of Primary Case

(a) Primary Case, age < 15 (b) Primary Case, age ≥ 25

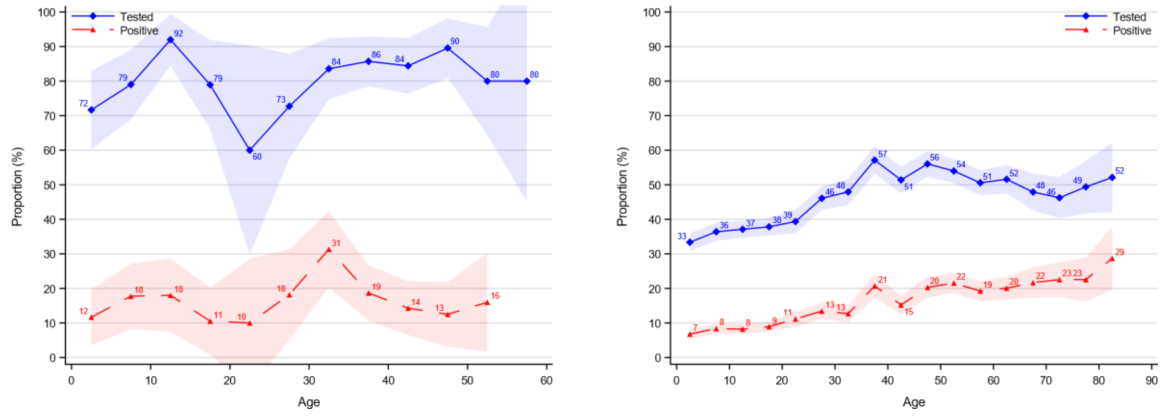

Notes: Shaded areas are 95% confidence bands clustered on the household level.

Figure S3: Age Structured Attack Rate and Transmission Risk

(a) Probability of being tested (b) Probability of being positive

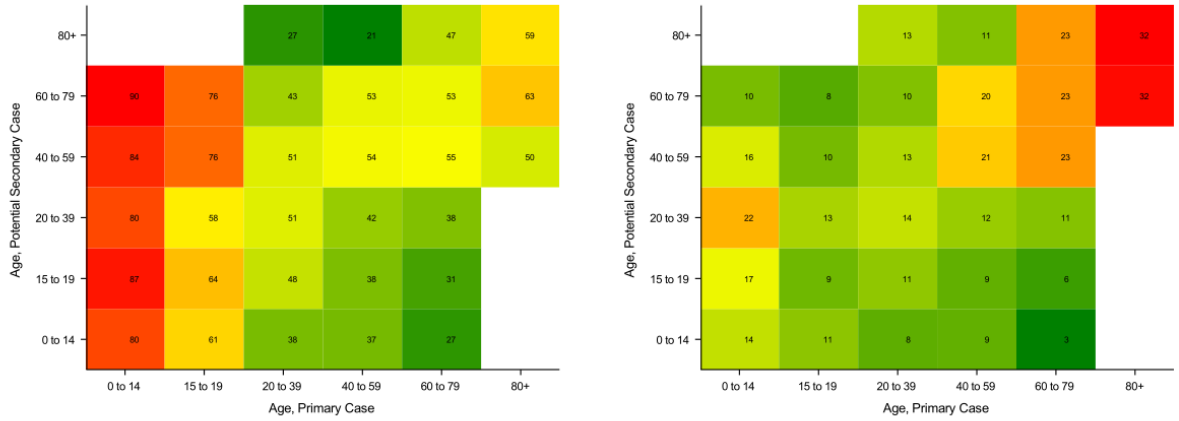

Notes: Panel a shows the probability of being tested and panel b the probability of having a positive test. The age of the primary case is depicted on the x-axis and the age of potential secondary cases on the y-axis.

B.1 Age structured Attack Rate by Sex

Figure S4: Age Structured Attack Rate by Sex

Sex of Primary Case

(a) Male

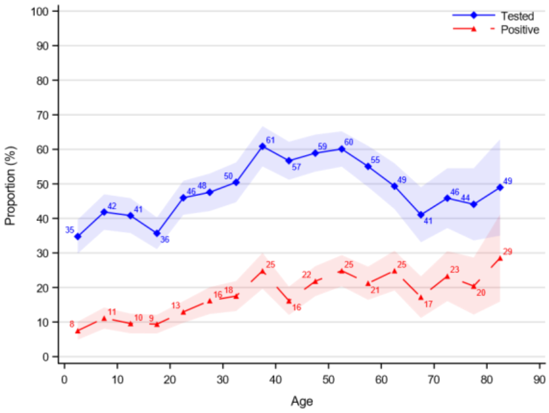

(b) Female

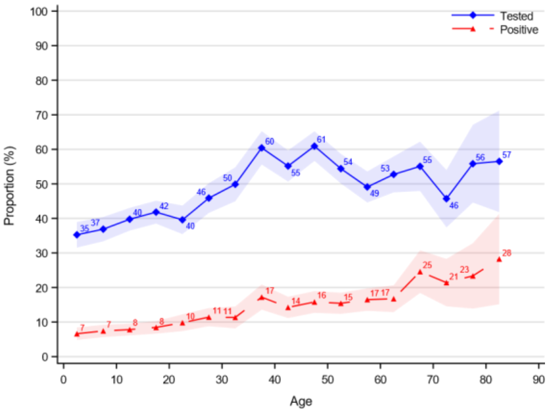

Sex of Potential Secondary Case

(c) Male

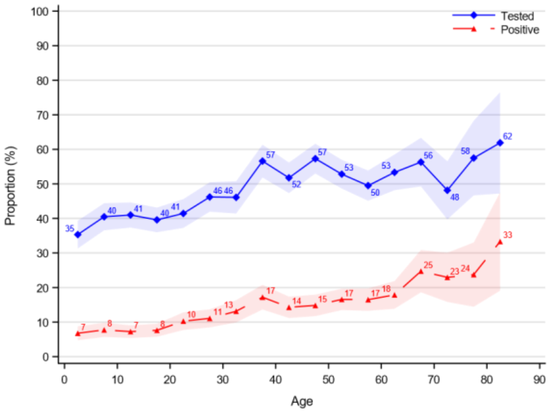

(d) Female

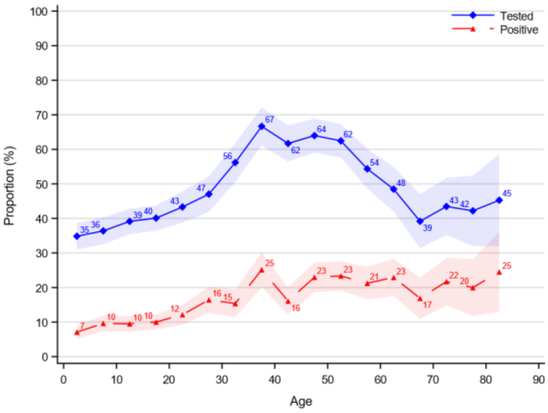

Sex of Potential Secondary Case, Primary Case age < 15

(e) Male

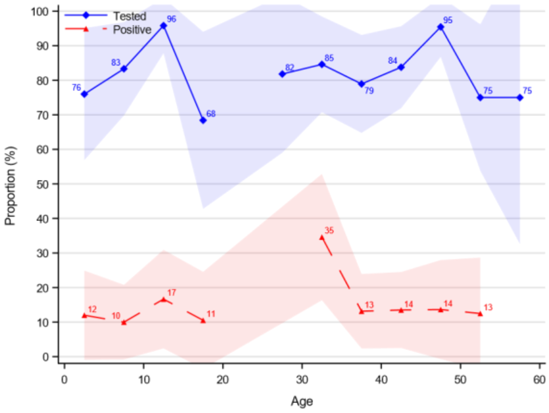

(f) Female

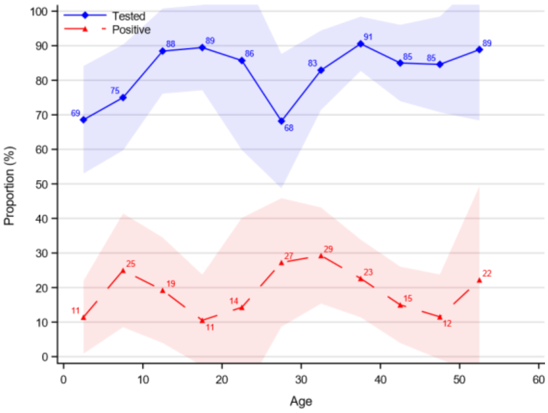

Notes: Shaded areas are 95% confidence bands clustered on the household level.

Supplementary Appendix C

Regression Estimates

Table S2: Regression Estimates: Attack Rate

	I	II	III	IV
Intercept	0.1336	0.1667	0.1556	0.1556
	(0.0035)**	(0.0099)**	(0.0102)**	(0.0123)**
Lockdown		-0.0578	-0.0569	-0.0538
		(0.0107)**	(0.0107)**	0.0133**
Early Reopening		-0.0095	-0.0078	-0.0123
		(0.0119)	(0.0119)	(0.0144)
Late Reopening		-	-	-
		(-)	(-)	(-)
Female			0.0224	0.0225
			(0.0057)**	(0.0161)
Female × Lockdown				-0.0068
				(0.0177)
Female × Early Reopening				0.0107
				(0.0194)
Number of Households	6,782	6,782	6,782	6,782
Number of Observations	14,220	14,220	14,220	14,220

Notes: Standard errors in parenthesis, clustered on the household level. *p<0.05, **p<0.01.

Table S3: Proportion of Cases by Household Size

		Number of Household Members				
		2	3	4	5	6
Number of cases	1	80.6	78.6	76.7	72.8	69.7
		(0.73)	(1.09)	(1.10)	(1.66)	(3.02)
	2	19.4	16.0	14.5	16.6	18.2
		(0.73)	(0.97)	(0.91)	(1.39)	(2.54)
	3		5.4	6.0	5.6	6.1
			(0.60)	(0.62)	(0.86)	(1.57)
	4			2.8	3.5	3.0
				(0.43)	(0.69)	(1.13)
	5				1.5	3.0
					(0.46)	(1.13)
	6					0.0
						(.)
Number of observations		5,87	4,263	5,916	3,58	1,386
Number of households		2,935	1,421	1,479	716	231

Notes: Estimates are in percentages. Standard errors in parenthesis, clustered on the household level.

Table S4: Proportion of Secondary Cases Per Infected Household

	Number of Secondary of Cases					
	0	1	2	3	4	5
	77.0	16.7	5.8	3.1	2.0	0.0
	(0.56)	(0.48)	(0.39)	(0.36)	(0.46)	(.)
Number of observations	21,015	21,015	15,145	10,882	4,966	1,386
Number of households	6,782	6,782	3,847	2,426	947	231

Notes: The estimates are calculated from households with at least one potential secondary case, i.e., households with two to six members. The estimate for two secondary cases is calculated from households with at least two potential secondary cases, i.e., households with three to six members. Estimates are in percentages. Standard errors in parenthesis, clustered on the household level.

Supplementary Appendix D

Robustness for Definition of Co-Primary Cases

Table S5: Robustness for Definition of Co-Primary Cases: Attack Rate

	I	II	III	IV	V
Excluding days ≤	None	0	1	2	3
Intercept	0.1667	0.1328	0.1133	0.0692	0.0453
	(0.0099)**	(0.0091)**	(0.0087)**	(0.0070)**	(0.0056)**
Lockdown	-0.0578	-0.0419	-0.0371	-0.0092	0.0034
	(0.0107)**	(0.0099)**	0.0094)**	(0.0077)	(0.0064)
Early Reopening	-0.0095	0.0027	0.0027	0.0224	0.0245
	(0.0119)	(0.0111)	(0.0105)	(0.0088)*	(0.0074)**
Late Reopening	-	-	-	-	-
	(-)	(-)	(-)	(-)	(-)
Number of Households	6,782	6,636	6,545	6,413	6,336
Number of Observations	14,220	13,871	13,610	13,271	13036

Notes: This table provides estimates on the robustness of the estimates for the attack rate depending on the definition of co-primary cases. Column I corresponds to column II in Table S5. In column II we exclude secondary cases that test positive on the same day (t =0) as the primary case. In column III, we exclude secondary cases that test positive within one day ($t \leq 1$) of the primary case. In column (IV), we exclude secondary cases that test positive within two days ($t \leq 2$) of the primary case. In column V, we exclude secondary cases that test positive within three days ($t \leq 3$) of the primary case. Standard errors in parenthesis, clustered on the household level. *p<0.05, **p<0.01.

Figure S5: Robustness for Definition of Co-Primary Cases: Age Structured Attack Rate

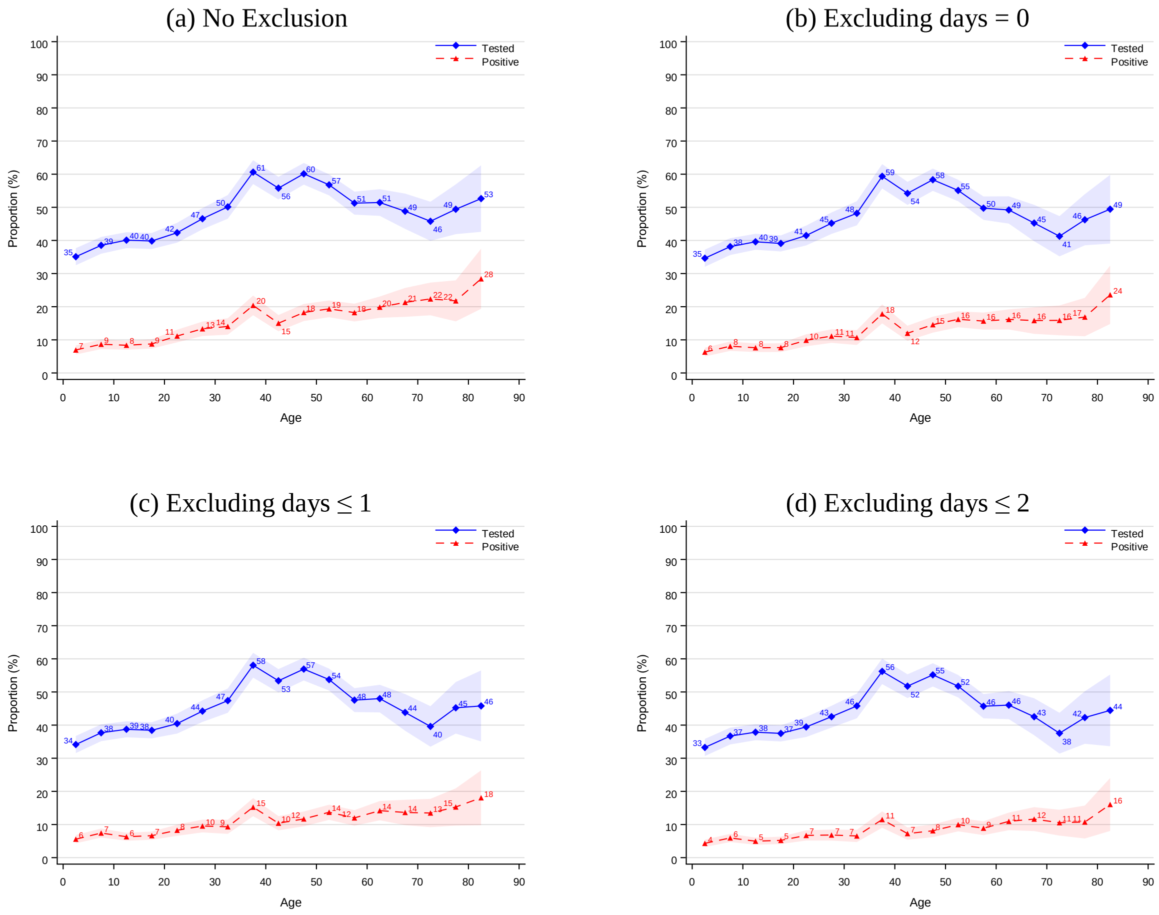

Notes: This figure illustrates robustness for the definition of co-primary cases with respect to the age structured attack rate. Panel a has no restrictions and is the same as figure 3. Panel b excludes secondary cases testing positive the same day as the primary case ($t = 0$). Panel c excludes secondary cases testing positive within 1 day of the primary case ($t \leq 1$). Panel d excludes secondary cases testing positive within 2 days of the primary case ($t \leq 2$).
